# A strain of *Phoma* species improves drought tolerance of *Pinus tabulaeformis*

**DOI:** 10.1038/s41598-021-87105-1

**Published:** 2021-04-07

**Authors:** Xiu Ren Zhou, Lei Dai, Gui Fang Xu, Hong Sheng Wang

**Affiliations:** School of Life Science and Technology, Henan Institute of Science and Technology, Xinxiang, 453002 China

**Keywords:** Microbiology, Applied microbiology

## Abstract

Global warming has led to frequent droughts, posing challenges for afforestation in arid and semiarid regions. In search of labor-saving and money-saving methods to improve the survival and growth rates of trees in these regions, we isolated and identified fungal endophytes that can potentially enhance the drought-resistance abilities of seedlings. We isolated 93 endophytic strains from the roots of *Pinus tabulaeformis* trees grown on an arid cliff. Three isolates increased the drought resistance of the tree seedlings. Using morphological, molecular, and physiological-biochemical methods, we identified three isolates as different clones of a strain of *Phoma* spp. and studied the strain’s effect on stress resistance-related substances in the seedlings. The results showed that the strain improved drought tolerance and increased the seedlings’ proline levels and antioxidant enzyme activities. The strain also secreted abundant extracellular abscisic acid, which likely triggered the seedlings’ protective mechanisms. This endophytic strain may provide a cheaper labor-saving, sustainable alternative to traditional methods of enhancing drought resistance.

## Introduction

Climate change and global warming cause frequent drought, presenting a serious threat to tree survival and growth^1,2^, which severely limits the development of artificial afforestation and environmental management in arid areas. Some sustainable, labor-saving, and money-saving alternatives to traditional methods, such as artificial irrigation and water retention agents, have become necessary. Much research has suggested that endophytic fungi can improve the ability of woody plants to resist drought stress. Therefore, screening for potential endophytes enhancing plant drought resistance may be a crucial step in finding a promising alternative to traditional methods.

Plants usually respond to drought stress through a series of morphological, physiological, and biochemical strategies, such as increasing their root biomass, decreasing their chlorophyll content, photosynthesis, stomatal conductance and leaf area, closing their stomata, and enhancing antioxidant activities^3–6^. Plant hormone levels also change, especially that of abscisic acid (ABA), which initiates protective mechanisms to protect plants from drought damage^7,8^. Endophytes can alter the morphological, physiological, and biochemical characteristics of plants to enhance their ability to adapt to a drought ecological environment^9–11^.

Endophytes can improve the host plant resistance to drought stress based on different mechanisms^9,12,13^. Some endophytes can increase the biomass, length, and number of host roots to enhance the ability of a plant to absorb water, thus improving its drought resistance^14–17^. Previous studies showed that inoculating gramineous plants with corresponding endophytic fungi significantly increased the plant tiller numbers, aboveground biomass, and leaf number, showing enhanced drought resistance compared with noninoculated plants^18,19^. Murphy et al. found that under drought stress, an endophytic fungus taken from *Hordeum murinum* subsp. *murinum* enabled the barley to preferentially allocate resources to its aboveground parts to achieve more aboveground biomass compared with uninoculated plants^20^. Under drought conditions, hybrid poplars inoculated with endophytic communities increased their antioxidant activities to achieve higher drought resistance than uninoculated plants^21^. Endophytic fungi improved the gas exchange, chlorophyll content, photosynthesis, and chloroplast fluorescence in both red oak and *Sorghum bicolor* seedlings to respond to drought stress^16,17,22,23^. Increased metabolism of amino acids, proteins, and other secondary substances has also been related to drought-resistance responses in some inoculated plants^22,24^. Levels of hormones, such as ABA, in some inoculated plants can be adjusted to improve their drought resistance^25–27^. Endophytes can also adjust a plant’s osmotic ability via active accumulation of organic and inorganic solutes (e.g., soluble sugars, glycine betaine, organic acids and proline) in the host tissue, thereby enhancing the plant’s drought resistance^28–31^. Therefore, an understanding of the mechanisms by which screened potential endophytes enhance plant drought resistance will promote the development and utilization of these endophytes.

In this study, we hypothesized that when *Pinus tabulaeformis*, a tree widely cultivated in northern and northwestern China, grows under extreme drought conditions, its roots are parasitized by endophytic fungi that can improve its drought resistance. Potential endophytic fungi strengthening *P. tabulaeformis* drought resistance were screened. The effects of these endophytes on seedling antioxidant activity, ABA content, and other physiological characteristics were measured and estimated. Finally, potential endophytic fungi improving plant drought resistance were obtained, and the mechanism through which they enhanced plant drought resistance was speculated.

## Results

We obtained 93 endophytic fungal isolates from Chinese pine roots. Screening of the drought-resistant endophytes revealed that all pine needles of the seedlings treated with fermentation broth supernatant (FBS) from strains PTD37, PTD56, and PTD78 remained green and tough after the application of 25% polyethylene glycol 6000 (PEG6000) solution. However, the other seedlings' early needles gradually lost their green color and became softer after PEG6000 application, suggesting that the seedlings watered with FBS from PTD37, PTD56, and PTD78 exhibited significantly improved drought-resistance abilities. The colonies, hyphae, ascospores, and perithecia of the three isolates all had the same morphological characteristics. The isolates also had identical nucleotide sequences in their internal transcribed spacer (ITS) regions (accession numbers: MN686446, MN686446, and MN686448) and their 18S rDNA (accession numbers: MN698959, MN698960, and MN698961). Therefore, the three isolates were likely clones from the same strain, and PTD37 was further studied and analyzed as a representative of the three isolates.

**Figure 1:**
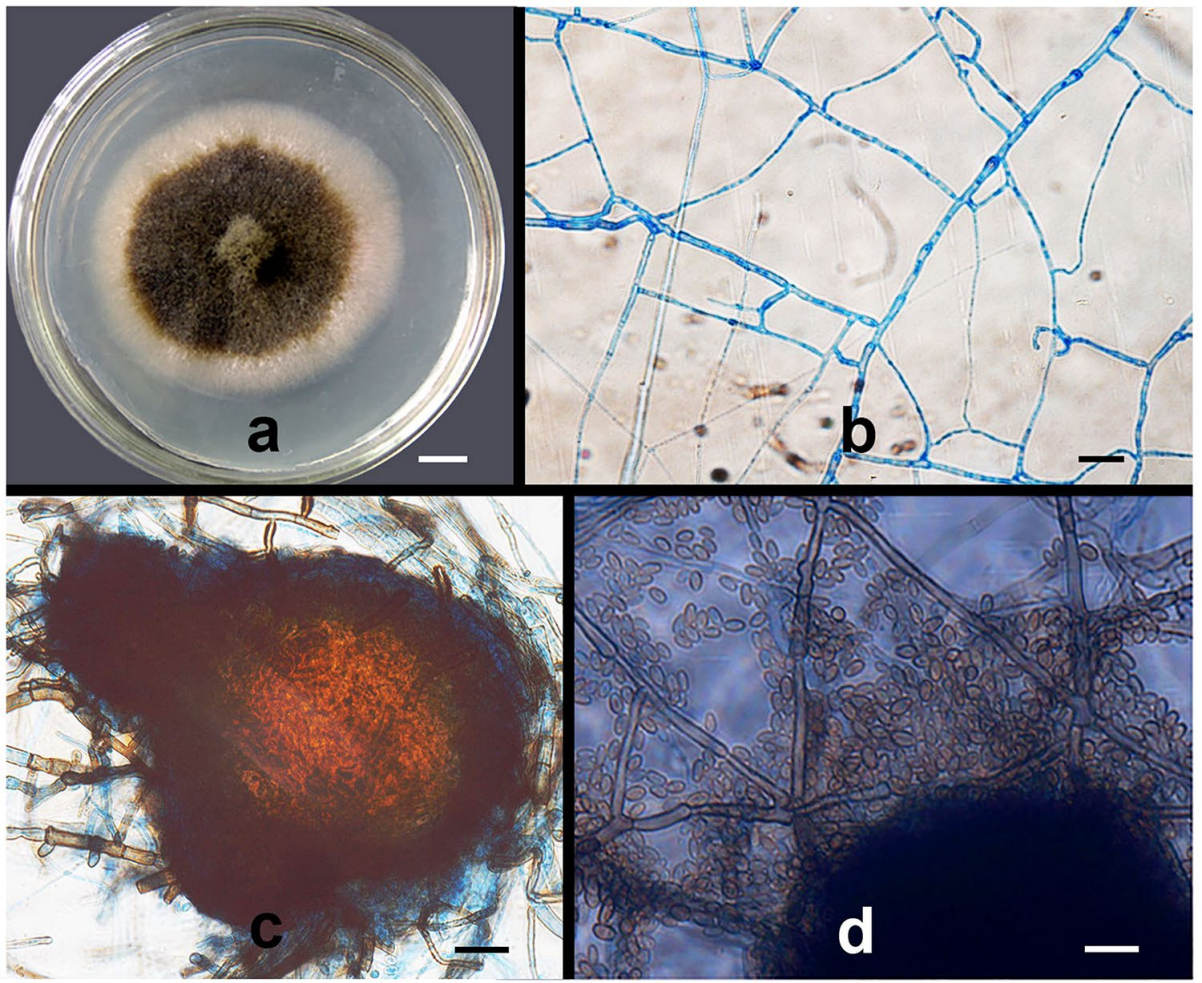
Morphological characteristics of strain PTD37 cultivated on PDA medium. (**a**) A colony after cultivation at 22–23 °C for 10 days. (**b**) Septate hyphae and branches. (**c**) Pycnidium. (**d**) Conidia. Scale bars: (**a**) = 1.0 cm; (**b**–**d**) = 10 µm.

Morphological and molecular analyses indicated that PTD37 was a species of *Phoma*. The PTD37 strain colonies grew slowly on potato dextrose agar (PDA) medium at 27–28 °C. The colonies were initially slightly almond colored; however, after 6 days, the colony centers became brown, while the margins remained slightly almond. The colony diameter averaged 5–6 cm on day 10 (Fig. 1a). The hyphae (0.5–3.0 μm wide) had typical septae, with a > 45° branching angle (Fig. 1b). Brownish black pycnidia (80–120 × 70–100) were produced during mycelial development (Fig. 1c). When the pycnidia matured, brown spores (4.5–6 × 2.5–3.0 μm) were released from the rupture once the perithecia were crushed and broken (Fig. 1d). These morphological features were similar to those of *Phoma* spp. described in the literature^32,33^. A comparison of the ITS sequence of PTD37 with entries in GenBank showed that the maximum score, total score, E-value, query cover, and percentage identity between the first three hits (accession numbers: MN944409.1, FJ755260.1, and FJ755259.1) and PTD37’s ITS were 907–909, 907–909, 99%, 0.0, and 99.40–99.60%, respectively. The sequences from the first three hits were from *Didymella macrostoma*, *Phoma medicaginis* strain CZ323, and *Phoma medicaginis* strain CZ316B. The PTD37 18S sequence comparison against known sequences in GenBank showed that the first three hits (accession numbers: KX519725.1, MT649541.1, and MT150595.1) were *Didymella macrostoma* isolate MS-2, *Didymella macrostoma* isolate 48, and *Phoma* sp. strain. The maximum scores, total scores, E-value, query cover, and percentage identity between the 18S of the first three hits and that of PTD37 were 913–918, 913–918, 99%, 0.0, and 99.02–99.41%, respectively. Therefore, the PTD37 isolate was identified as *Phoma* spp.

**Figure 2:**
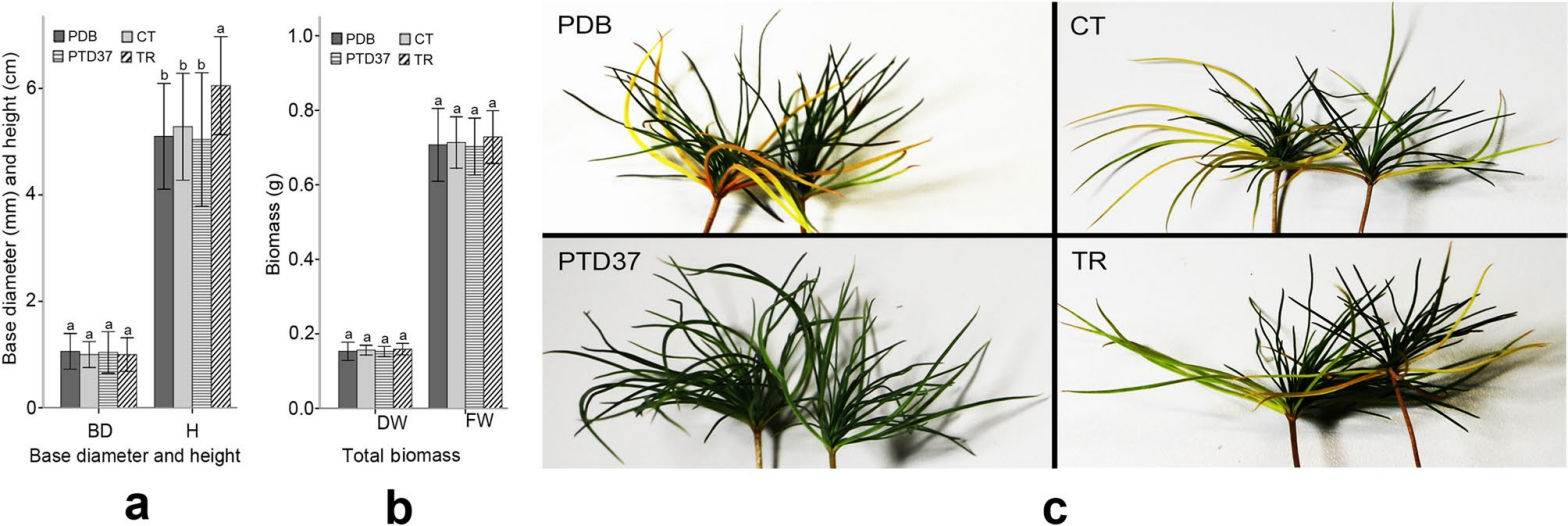
Effect of different treatments on total biomass and seedling phenotype before PEG application. (**a**) Effect of different treatments on the basal diameter and height of seedlings before PEG application. (**b**) Effect of different treatments on the total biomass of seedlings before PEG application. (**c**) Vegetative appearances of different treatment seedlings 15 days after PEG application, showing the seedlings treated by PTD37 with green and tough leaves but other seedlings with a loss of green color and wilted lower leaves. *BD *basal diameter, *H *height, *CT *control, *DW *dry biomass, *FW *fresh weight, *PDB *potato dextrose broth, *PTD37 *fermentation broth supernatant of the PTD37 strain, *TR *fermentation broth supernatant of *Trichoderma harzianum*. In (**a**) and (**b**), data are the means ± SD from five independent tests. Different letters on error bars indicate significant differences at *P* = 0.05 between the analyzed traits of seedlings from different treatments before PEG application. Scale bars: (**c**) = 5.0 cm.

After application of the PTD37 FBS to the seedlings, their total biomass, height, and ground diameter did not significantly differ from those of the other treatments except the *Trichoderma harzianum* FBS treatment (Fig. 2a,b). After treatment with *T. harzianum* FBS, the seedlings were significantly taller than the other seedlings. However, all seedlings had similar total biomasses and ground diameters (Fig. 2a,b). After application of PEG6000 solution for 15 days, the seedlings pretreated with PTD37 FBS had green and tough leaves, whereas the lower leaves of the seedlings that received the other treatments were yellow-green, yellow, or brown and softer (Fig. 2c).

**Figure 3:**
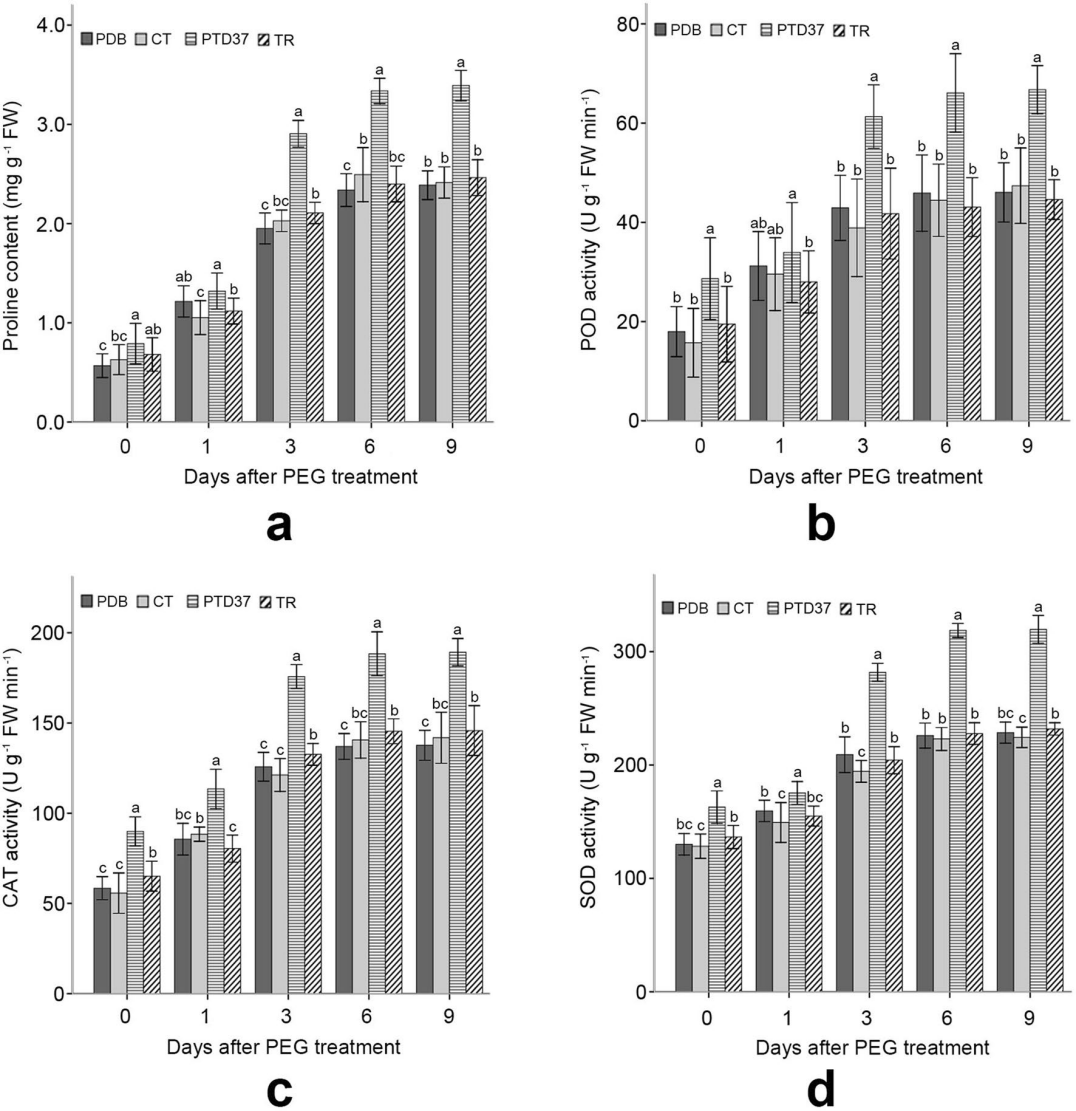
Effect of different treatments on the proline content and POD, CAT and SOD activities of seedling leaves before and after PEG application. (**a**) Effect of PEG application on the leaf proline content of seedlings under different treatments. (**b**) Effect of PEG application on the leaf POD activity of seedlings under different treatments. (**c**) Effect of PEG application on the leaf CAT activity of seedlings under different treatments. **(d)** Effect of PEG application on the leaf SOD activity of seedlings under different treatments. CT = control; PDB = potato dextrose broth; PTD37 = fermentation broth supernatant of the PTD37 strain; TR = fermentation broth supernatant of *T. harzianum*. Data are the means ± SD from five independent tests. Different letters on error bars indicate significant differences at *P* = 0.05 between analyzed traits of seedlings from different treatments on the same day after PEG application. The values on day 0 after PEG treatment indicate that they were measured before PEG application.

PTD37-treated seedlings had a significantly higher proline level than the seedlings in the potato dextrose broth (PDB) and distilled water (CT) treatments before PEG application, but there was no significant difference between PTD37 and TR. Application of PTD37 FBS significantly increased the seedlings’ proline levels compared with those of the other seedlings after PEG application (Fig. 3a). The peroxidase (POD), catalase (CAT), and superoxide dismutase (SOD) activities of the PTD37-treated plants were more robust than those of the plants that received the other treatments during the measurement period (Fig. 3b–d). Once treated with PEG solution, the proline content and antioxidant enzyme activities of all seedlings increased rapidly over the first 6 days, especially over the first 3 days (Fig. 3). Treatment with PEG gradually increased the difference in the proline content and antioxidant enzyme activity between the PTD37-treated seedlings and the other seedlings (Fig. 3). These levels also reached or neared the maximum on day 6 after PEG application but only increased slightly or did not change from days 6–9.

**Figure 4:**
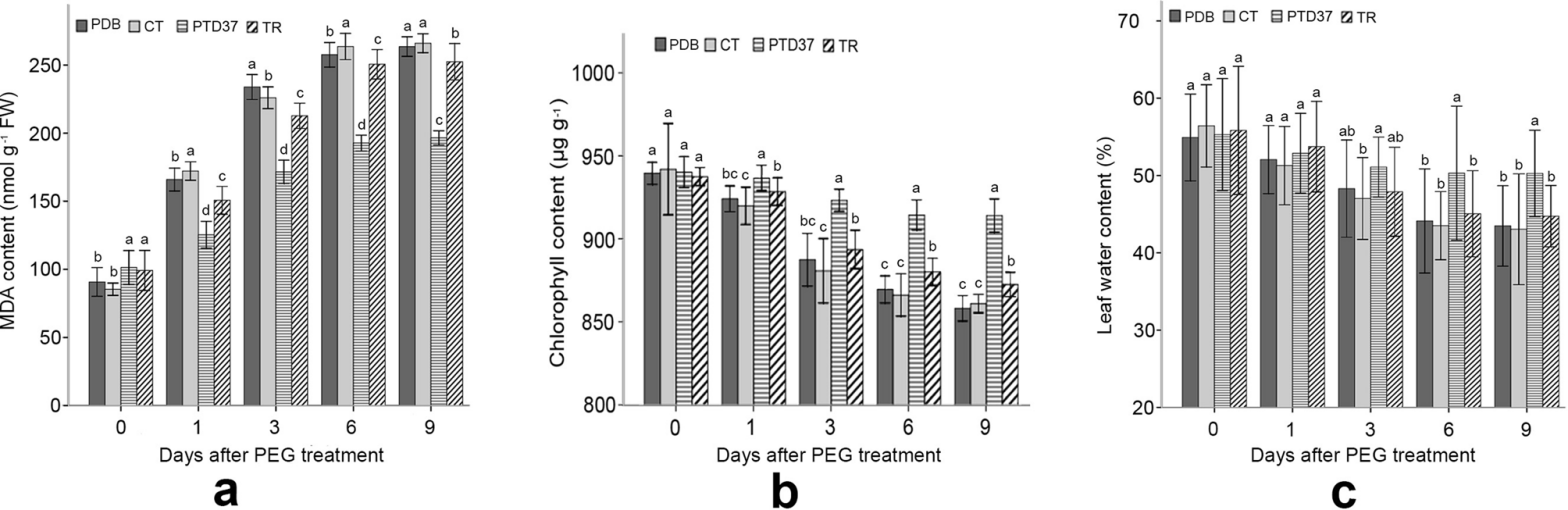
Effect of different treatments on the MDA, chlorophyll, and water contents of seedling leaves before and after PEG application. (**a**) Effect of PEG application on the leaf MDA content of seedlings under different treatments. (**b**) Effect of PEG application on the leaf chlorophyll content of seedlings under different treatments. (**c**) Effect of PEG application on the leaf water content of seedlings under different treatments. *PDB *potato dextrose broth, *PTD37 *fermentation broth supernatant of the PTD37 strain, *TR *fermentation broth supernatant of *T. harzianum*. Data are the means ± SD from five independent tests. Different letters on error bars indicate significant differences at *P* = 0.05 between the analyzed composition contents of seedlings from different treatments on the same day after PEG application. The values on day 0 after PEG treatment indicate that these values were measured before PEG application.

Before PEG application, no seedlings differed in their chlorophyll or leaf moisture content, but they did differ in their malonic dialdehyde (MDA) content (Fig. 4). However, after PEG application, the PTD37-FBS-treated seedling leaves had higher chlorophyll and water contents and lower MDA levels than the leaves of the other treatments. Thus, PTD37-FBS-treated seedlings could better withstand drought-stress conditions (Fig. 4). After PEG application, the chlorophyll content in the leaves of the seedlings treated with *T. harzianum* FBS was higher than that in those treated with distilled water or PDB FBS but was significantly lower than that in the PTD37–FBS-treated seedling leaves (Fig. 4b). The MDA, chlorophyll, and water contents in all treatments stopped changing 6–9 days after PEG application (Fig. 4a).

**Figure 5:**
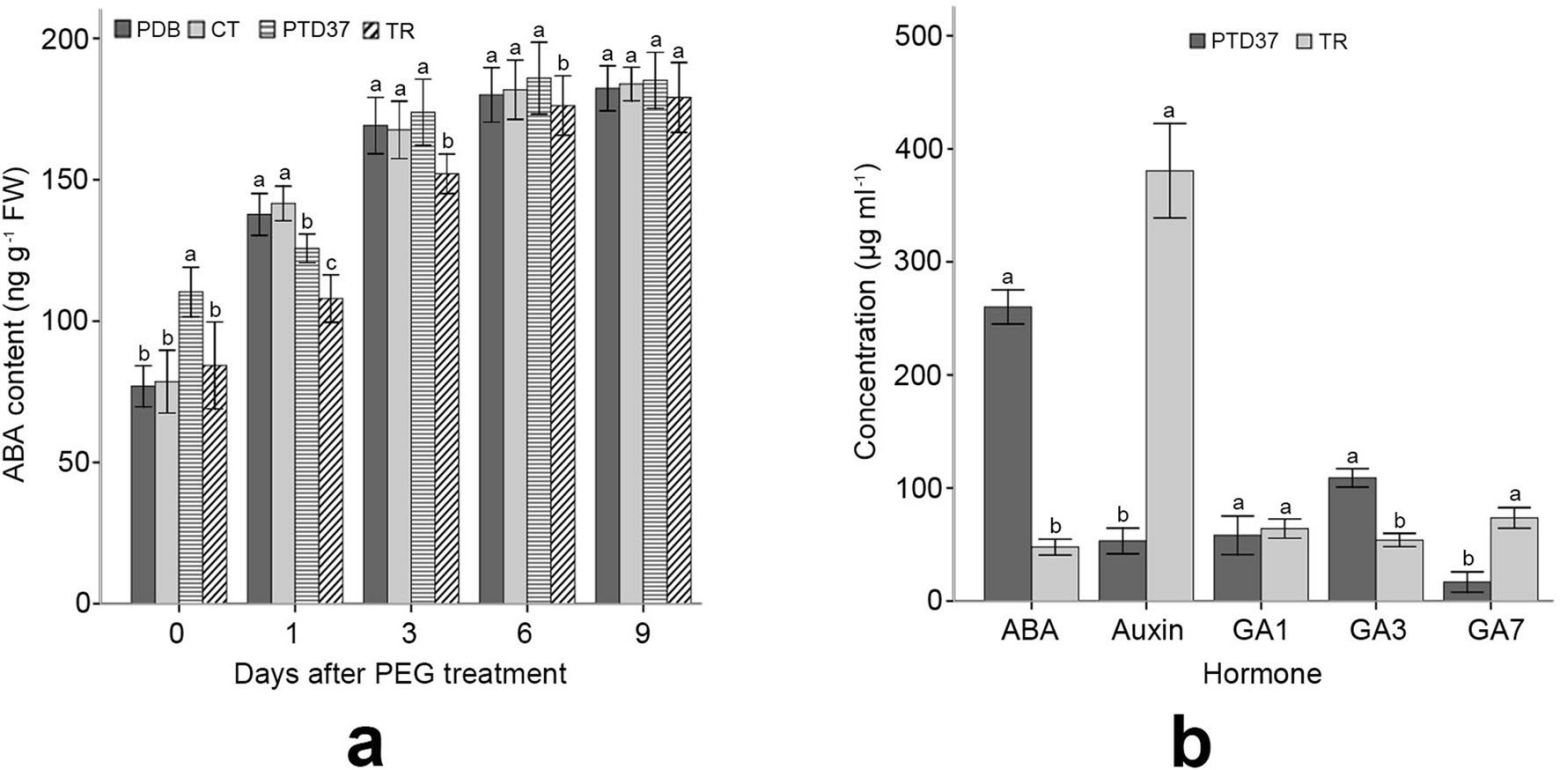
Effect of PEG application on the leaf ABA content of seedlings under different treatments and the levels of different hormones in PTD37 and *T. harzianum* fermentation broth. (**a**) Effect of PEG application on the leaf ABA content of seedlings under different treatments. (**b**) Levels of ABA, auxin, GA1, GA3 and GA7 in PTD37 and *T. harzianum* fermentation broth. PDB = potato dextrose broth; *PTD37 *fermentation broth supernatant of the PTD37 strain, *TR *fermentation broth supernatant of *T. harzianum*. Data are the means ± SD from five independent tests. Different letters on error bars indicate significant differences at *P* = 0.05 between the ABA contents of seedlings from different treatments on the same day after PEG application. They also indicate significant differences at *P* = 0.05 between the analyzed hormone levels of different fungal fermentation broths. The values on day 0 after PEG treatment indicate that they were measured before PEG application.

The PTD37-FBS-treated seedlings had higher ABA levels than the other seedlings before PEG application. However, the ABA content of the PTD37-treated seedlings increased significantly slower than that of the control, PDB-treated or CT-treated seedlings on day 1 after PEG treatment (Fig. 5a), indicating lower stress perception in the PTD37-treated plants owing to their better stress resistance. ABA levels in the PTD37-treated plants became progressively similar to those of the other plants from day 3 of PEG application (Fig. 5a). The ABA level in the PTD37 FBS was approximately 7 times that of the *T. harzianum* FBS*.* Gibberellin (GA) 3 was also significantly higher in the PTD37 FBS than in the *T. harzianum* FBS. The auxin level of *T. harzianum* FBS was 7 times that of PTD37 FBS. *T. harzianum* FBS had a significantly higher level of GA7 than PTD37 FBS. However, these two FBSs had similar GA1 contents (Fig. 5b).

## Discussion

To ensure the survival of *P. tabulaeformis* in semiarid and arid areas, irrigation and water retention agents require substantial financial resources, which severely limits artificial afforestation and environmental management. Endophytic fungi that improved host plant drought resistance are considered to be a sustainable, labor-saving, and money-saving approach. Whether *P. tabulaeformis* is parasitized by fungal endophytes, enhancing its drought resistance, remains unclear. The screening of potential endophytes enhancing the ability of *P. tabulaeformis* to resist drought and research on the interaction mechanism between them will facilitate the development of an alternative to traditional methods.

In this study, we isolated a fungal endophyte from the roots of *P. tabulaeformis*, which can potentially improve the ability of this species to resist drought. Previous researchers screened a variety of fungal endophytes that can improve plant drought resistance. These endophytes may belong to different fungal taxa. Based on morphological and molecular data, the current isolate was identified as a species of the genus *Phoma*. In the past, no species improving plant stress resistance was found in this genus. The species in the genus *Phoma* are generally pathogenic fungi and often cause decay or wilting in the host plant^34,35^. Our findings may partly support a hypothesis that the harmful parasitic relationship between specific pathogenic microorganisms and their host plants can become mutually beneficial relationships through coordinated evolution^36,37^.

The current study revealed that the PTD37-treated *P. tabulaeformis* seedlings had significantly higher antioxidant activity than other treatments. Plants quickly accumulate reactive oxygen species under abiotic stresses, which are often scavenged by the antioxidants they produce to prevent damage to their tissues and organs^38–40^. Some researchers have demonstrated that symbiotic endophytes can promote the production of antioxidant compounds in plants to improve plant abiotic stress tolerance^21,41,42^. *Piriformospora indica* was also found to upregulate antioxidant activities in inoculated plants to enhance salt^43^ and drought resistance^44^. These reports are consistent with our finding that PTD37-treated seedlings showed higher drought tolerance than seedlings in other treatments. It is proposed that antioxidant activities may be a major mechanism of improvement of drought resistance in *P. tabulaeformis* by PTD37 FBS.

Our study showed that the chlorophyll, water, and MDA contents in the PTD37-treated seedlings were altered less than those in the other treatments after PEG application. Drought causes plant chlorophyll degradation, MDA accumulation, and water loss in tissues, which result in a decline in photosynthesis^45–47^. However, drought-tolerant plants can reduce chlorophyll degradation, MDA accumulation, and water loss compared with drought-sensitive plants. Plants inoculated with certain endophytes can maintain higher water and chlorophyll contents and lower MDA levels than noninoculated plants under drought stress, suggesting that these microbes can enhance plant drought resistance^16,17,22,23,28–30^. Similar to the above reports, *P. tabulaeformis* seedlings treated with PTD37 accumulated less MDA, broke down less chlorophyll, and lost less tissue water than the plants in other treatments. This result indicated that PTD37 may play a role in the improvement of chlorophyll, MDA, and water contents under drought stress.

The PTD37 fermentation broth contained high levels of ABA in the present study. ABA is considered a crucial signaling substance for plant stress-tolerance signaling pathways^48–50^. Drought can trigger ABA formation, causing stomatal closure and decreasing photosynthesis^51–54^. It has been demonstrated that application of exogenous ABA can induce a plant’s drought tolerance by increasing enzymatic and nonenzymatic antioxidant production^55–57^. The application of broth containing ABA secreted by PTD37 yielded similar results to that of exogenous ABA. Plants treated with the PTD37 fermentation broth showed a higher drought tolerance than plants in other treatments. Therefore, ABA in PTD37 fermentation broth may trigger a series of drought-resistance responses in seedlings to enhance drought resistance.

A primary response of plants under drought stress is proline accumulation, which weakens the damage caused to tissues and cells^30,45^. Proline accumulation is considered to protect membranes and proteins from damage by reactive oxygen species and to play a role in osmotic adjustment under abiotic stress^28,30,45^. Our study showed that the seedlings in the PTD37 treatment had a higher proline content than those in the other treatments. Some researchers have shown that plants inoculated with endophytes can produce less proline than noninoculated plants under drought conditions^16,23^. However, more researchers have demonstrated that inoculated plants have higher levels of proline than noninoculated plants under drought conditions^23,24,28,29,46^. The results of the current study are consistent with the conclusions of most published literature. After PEG application, PTD37-treated seedlings were more drought resistant than other seedlings. This may suggest that the enhanced proline production in PTD37-treated seedlings may reduce the damage to cells caused by drought.

Endophytes can enhance the ability of plants to resist drought by increasing plant total biomass, aboveground or belowground biomass, tiller number, root length, and root number^14–20^. In this study, the basal diameter, height, and total biomass of PTD37-treated seedlings were not significantly higher than those of other treatments. This may imply that PTD37 does not strengthen a seedling’s drought tolerance through biomass, height, and basal diameter. However, we did not measure the aboveground and belowground biomass, so it is not clear whether PTD37 affects biomass allocation to improve seedling drought tolerance.

In the current research, a potential endophytic fungus enhancing *P. tabulaeformis* drought tolerance was isolated, and the mechanism by which FBS enhances the drought resistance of seedlings was studied. This will facilitate the development of an alternative to traditional methods for drought resistance of *P. tabulaeformis* and will provide a better understanding of the mechanism by which fungal endophytes improve plant drought resistance. However, there is still much work to be done in the future. Potential endophytes should be inoculated into *P. tabulaeformis*, and the mechanism by which endophytes strengthen plant drought resistance requires further research. The molecular basis of the relationship between the endophyte and plants also needs to be studied.

## Materials and methods

### Materials

This experimental study complies with Chinese national and local laws, and sample collection has been permitted by the local relevant administration. The root samples used to isolate the endophytes were collected from three mature Chinese pine trees grown on the top of a south-facing arid cliff 1300 m above sea level in the Taihang Mountains. Each tree provided 16 root segments (20–30 mm long, 3–5 mm diameter), which were taken from roots at different positions. After collection, the samples were immediately rinsed with distilled water, placed in a sealed fresh-keeping bag, and stored in a refrigerator at 4 °C. Voucher specimens (ID: TH2017022101-6) for the trees were deposited at the Herbarium of Henan Institute of Science and Technology and were identified by Dr. Pengming Yang.

### Reagents

Analytical-grade reagents were used to determine the POD, CAT, SOD, MDA, proline, and chlorophyll levels (Sangon Biotech [Shanghai] Co., Ltd. or Solarbio Science and Technology [Shanghai] Co., Ltd.). All deuterated standards and calibration samples for determining the ABA, auxin, GA1, GA3, and GA7 levels were purchased from Sigma-Aldrich (St. Louis, MO, USA). Reagents for the high-performance liquid phase were sourced from Thermo Fisher Scientific Inc. Consumables such as petri dishes, agar, and sucrose for endophytic isolation and fermentation were purchased from Solarbio Science and Technology Co., Ltd. PDA, PDB, or water agar (WA) media were used. The PDB medium contained 200 g l^−1^ potato and 20 g l^−1^ d-glucose. The PDA medium contained 200 g l^−1^ potato, 20 g l^−1^ d-glucose and 15 g l^−1^ agar. The WA medium was prepared by adding 10 g agar to 1000 ml distilled water.

### Endophytic isolation

The Chinese pine root samples were thoroughly rinsed with sterilized distilled water, and their surfaces were washed with a clean brush to remove the soil particles and other impurities on the roots. The surface-cleaned roots were sterilized in 75% alcohol for 4 min and then cut into small pieces (~ 5 mm long). The pieces were sterilized in 0.1% mercury chloride solution for 5 min and then rinsed with sterile distilled water for 3 min to remove residual mercuric chloride, as per Li et al. (2015)^58^. The sterilized root pieces were then placed on 1.0% WA medium and cultivated in the dark at 22–23 °C. Three blank controls containing only WA were cultivated under the same conditions. All cultivars were carefully observed daily. After 3–5 days, white mycelia or hyphae of the endophytes emerged on the root piece surfaces. These hyphae were transferred to PDA medium for further cultivation. Hyphae that arose on the newly positioned root pieces were isolated and cultured to obtain as many different endophytic strains as possible. After 1.5 months, most endophytes growing on the WA medium were isolated. Primary isolates were further purified to ensure a single strain in each medium.

### Preparation of the FBS of the isolates and *T. harzianum*

Each isolate was inoculated in 200 ml PDB medium in a triangular flask and then cultured in a 150-rpm shaker at 25 °C for 10 days. Afterward, this fermentation broth was centrifuged at 7000×*g* for 30 min, and 100 ml of supernatant was taken for the following tests. *T. Harzianum* was fermented under the same conditions, and its FBS was obtained. *T. harzianum* and other *Trichoderma* spp. can enhance abiotic and biotic stress resistance in plants^59–62^.

### Screening of the isolates enhancing the drought-stress tolerance of Chinese pine seedlings

Chinese pine seeds were disinfected, soaked and germinated. After radicles emerged from ~ 80% of the seeds, every 10 germinated seeds were sown in a square plastic plant pot (12 cm wide, 10 mm high) containing a sterile mixture of river sand and perlite (2: 1 v/v) that was autoclaved at 125 °C for 45 min. Next, 562 pots were prepared, numbered, and transferred to a greenhouse at 23–25 °C with 60–70% relative humidity. When all seedlings emerged, they were watered with 2 ml/plant of Hogland’s nutrient solution every 5 days and were sprayed with distilled water to prevent drought. After ~ 20 days, when the seedlings grew approximately 20 needles, 279 treatments were completely randomly assigned to 558 of the 562 pots. The treatments comprised three FBS dilutions (twofold, fivefold, and tenfold) of each of the 93 isolates, with two replicates per treatment. The remaining 4 pots were used as controls and were watered with 3 ml distilled water per plant every 3 days. The other seedlings were treated with 3 ml diluted FBS from each relevant isolate per plant every 3 days. After 21 days, each pot was treated with 50 ml 25% PEG6000 solution except that 2 of 4 control pots were treated with 50 ml distilled water. After 15 days, the plants with enhanced drought tolerance were differentiated based on their morphological characteristics, such as leaf color and wilting degree, and the strain that best promoted drought resistance in Chinese pine was determined.

### Experimental design of the effect of the target fungus (PTD37 strain) on Chinese pine seedling physiological and biochemical characteristics

Chinese pine seeds were sterilized, germinated, and planted in pots, as described above. Then, 120 pots of seedlings with ~ 20 needles were prepared and randomly combined into 20 experimental units, each consisting of 6 completely randomly selected pots. Five pots were then independently assigned to each of four treatments as follows: application of 3 ml fivefold PTD37 FBS per plant, application of 3 ml fivefold *T. harzianum* FBS per plant, application of fivefold PDB medium FBS per plant, and application of 3 ml distilled water per plant. Afterward, seedlings from each experimental unit were treated with 3 ml fivefold FBS or distilled water per plant every 3 days according to the experimental design. After 21 days, all seedlings were treated with 5 ml 25% PEG6000 solution per plant.

### Measurement of the seedling basal diameter and height

Before application of PEG6000 solution, the seedling basal diameters and heights were measured to evaluate the different treatment effects. Three plants from each treatment were selected randomly and measured with a digital Vernier caliper. This measurement was repeated three times.

### Determination of the total fresh biomass, dry biomass, and leaf water content

Before application of PEG6000 solution, three plants per treatment were randomly selected to measure the total fresh biomass using a 0.0001-mg electronic analytical balance. This measurement was repeated three times. The plants were similarly sampled again, oven-dried at 70 °C for 72 h and weighed. After PEG application, plants of different treatments were sampled every 3 days. Three seedlings were randomly sampled and weighed immediately; this was repeated three times. The samples were oven-dried at 70 °C for 72 h and weighed. The leaf water content was determined based on the leaf fresh and dry weights.

### Leaf sample collection to determine the physiological and biochemical characteristics of the seedlings

After treatment for 21 days, the heights and basal diameters of 8 randomly selected plants per replication were measured. The needles of 8 randomly selected plants per replication were collected and immediately stored in a refrigerator at − 80 °C to determine the antioxidant compound and enzyme activity levels. On days 1, 3, 6, and 9 after PEG treatment, the leaf samples were collected and stored to determine the compound levels and enzyme activities as described above.

### Estimation of SOD, POD, and CAT activities in Chinese pine seedling leaves

SOD, POD, and catalase activities were determined according to Sunkar (2010)^59^and Zhang (2011)^60^.

#### Estimation of SOD activity

Chinese pine leaves (0.2 g) stored at − 80 °C were added to a mortar containing 2 ml of 0.05 M phosphate-buffered saline (PBS: pH 7.8) in an ice bath and quickly ground. Then, 8 ml PBS was transferred into this mortar to obtain a 10-ml final volume. Next, 5 ml of ground leaf tissue mixture was transferred into a 5-ml centrifuge tube and centrifuged at 12,000 rpm for 10 min, and then 50 μl of supernatant was placed in a new centrifuge tube. Next, 300 μl 130 μM methionine, 300 μl 750 μM nitrotetrazolium blue chloride, 300 μl 100 μM EDTA-Na2 solution, 300 μl 20 μM riboflavin solution, and 250 μl distilled water were added to this tube in sequence to obtain a 3-ml final volume. The test tube was placed under a 4000 l × fluorescent lamp at 24 °C for 20 min; an enzyme-free reaction system used as the control. The absorbance of this system was measured at 560 nm.

#### Estimation of POD activity

Chinese pine leaves (0.2 g) stored at − 80 °C were placed in a mortar with 2 ml of 100 mM PBS buffer (pH 6.0) in an ice bath and quickly ground. Then, 8 ml PBS was added to the mortar to obtain a 10 ml final volume. The ground tissue mixture (5 ml) was transferred to a 5-ml centrifuge tube and centrifuged at 12,000 rpm for 10 min. The supernatant was transferred into a 20-ml volumetric flask and brought to a total volume of 20 ml with PBS buffer (pH 6.0). Next, 0.1 ml of the sample solution was transferred into a test tube containing 2.3 ml of PBS, 0.5 ml 5 mM guaiacol and 0.1 ml 4 mM hydrogen peroxide solution. The absorbance of the reaction system was measured every 30 s at 470 nm. A reaction system containing no sample was used as a control.

#### Estimation of CAT activity

Chinese pine leaves (0.2 g) stored at − 80 °C were ground in a mortar containing 2 ml 0.05 M PBS (pH 7.0) in an ice bath and 8 ml PBS was added to obtain a 10-ml final volume. Next, 5 ml of sample solution was added to a 5-ml centrifuge tube and centrifuged at 5000 rpm in a refrigerated centrifuge for 20 min. Afterward, 0.2 ml of supernatant was transferred into a test tube and mixed with 1.5 ml PBS and 1 ml distilled water. Next, 0.3 ml of 0.1 M hydrogen peroxide solution was added to this tube. The absorbance of the reaction system was measured immediately at 240 nm and then every minute for 4 min. Enzyme-denatured reaction systems were used as controls.

### Valuation of MDA, proline, and chlorophyll contents in Chinese pine seedling leaves

The MDA, proline, and chlorophyll contents were determined following Sunkar (2010)^63^ and Zhang (2011)^64^ with slight modifications.

#### Measurement of the MDA content

Chinese pine leaves (0.2 g) stored at − 80 °C and 2 ml 10% trichloroacetic acid (TCA) were mixed and ground in a mortar in an ice bath, and 8 ml of 10% TCA was added to the mortar to obtain a 10-ml final volume. Next, 5 ml of sample solution was transferred to an Eppendorf tube and centrifuged at 5000 rpm in a refrigerated centrifuge for 20 min. Then, 1 ml of the supernatant was taken and mixed with 3 ml 0.6% 2-thiobarbituric acid. The mixed solution was heated in a boiling bath for 20 min, rapidly cooled in an ice bath, and centrifuged at 1000 rpm for 5 min. Afterward, the supernatant was taken, and its absorbance was estimated at 532, 600, and 450 nm.

#### Measurement of the proline content

Chinese pine leaves (0.2 g) stored at − 80 °C were ground to a slurry in a mortar with 2 ml of 3% 5-sulfosalicylic acid dehydrate, and then 8 ml 3% 5-sulfosalicylic acid dehydrate was transferred into this mortar. The sample solution (5 ml) was added to a centrifuge tube, and then heated and shaken in a boiling water bath for 10 min. The solution was then cooled in a 20 °C water bath and centrifuged at 5000 rpm for 10 min. Then, 1 ml of the supernatant was transferred to a 10-ml test tube with a lid. Next, 2 ml glacial acetic acid and 2 ml acid ninhydrin were added to this tube, which was sealed with a lid to prevent evaporation, and then heated in boiling water for 30 min. After cooling in a 20 °C water bath, the solution was mixed with 4 ml toluene, shaken for 30 s, and then left to stand for 2 h. Finally, 0.5 ml of the red proline toluene solution, located at the top of the system, was carefully removed, and its absorbance was read at 520 nm.

#### Measurement of the chlorophyll content

Chinese pine leaves (0.2 g) stored at − 80 °C were ground in a mortar containing 2 ml ethanol, and then 8 ml ethanol was added. The sample in the mortar was transferred into a 15-ml plastic test tube, and then the sample solution was fully oscillated on a vortex oscillator and filtered. The filtrate was brought to a 50-ml total volume with ethanol, and its absorbance was measured at 710, 665 (chlorophyll *a*), and 649 nm (chlorophyll *b*). The extinction coefficients of chlorophyll *a* and *b* were 72.23 l g^−1^ cm^−1^ and 38.52 l g^−1^ cm^−1^, respectively, when the spectrophotometer was zeroed at 710 nm.

### Detection of the hormone content

Hormone levels in the fungal fermentation broth and Chinese pine leaves were determined as per Pan et al. (2010)^65^.

#### Detection of ABA, auxin, GA1, GA3, and GA7 levels in endophytes and *T. harzianum* fermentation broth

Fermentation broth (5 ml) was centrifuged at 6000 rpm for 10 min; 1.9 ml of supernatant was added to a 5-ml centrifuge tube, and then 100 μl deuterated hormone internal standard and 2 ml dichloromethane were added. The tube was shaken on a constant temperature shaker oscillator at 4 °C for 30 min. The mixed solution was centrifuged at 12,000 rpm at 4 °C for 5 min. The bottom layer solution (900 μl) was taken, transferred to a 2-ml centrifuge tube and evaporated in a nitrogen evaporator until a light-brown wet cream formed. The cream was dissolved in 200 μl methanol. Finally, 25 μl of sample solution was injected into a C18 Gemini column (Phenomenex USA) for HPLC–ESI–MS/MS analysis.

#### Detection of ABA contents in seedlings leaves

Chinese pine leaves (0.2 g) stored at − 80 °C were ground into powder in liquid nitrogen in a mortar, and then 100 mg of the powder was transferred into a 5-ml centrifuge tube. Next, 100 μl ABA deuterated internal standard and 1 ml extraction solution were added to the tube and incubated in a shaker at 100 rpm at 4 °C for 30 min. Afterward, 2 ml dichloromethane was added to the solution, shaken on a constant temperature shaker oscillator at 4 °C for 30 min, and then centrifuged at 12,000 rpm at 4 °C for 5 min. Next, 900 μl of solution from the lower layer was transferred to a 2-ml Eppendorf tube and evaporated to form a wet cream in a nitrogen evaporator. The cream was mixed with 200 μl methanol and dissolved. Finally, 25 μl of sample solution was injected into a C18 Gemini column (Phenomenex USA) for HPLC–ESI–MS/MS analysis.

### Morphological and molecular identification of the endophytic fungi

After culturing the isolates in PDA medium at 27–28 °C for 7–15 days, the hyphae, mycelium, conidia, and conidiophores were observed and photographed with an optical microscope. Endophytic DNA was extracted as per Raeder and Broda (1985)^66^. The ITS (ITS1 5′-TCCGTAGGTGAACCTGCGG-3′; ITS2 5′-TCCTCCGCTTATTGATATGC-3′) and 18S (NS1 5′-GTAGTCATATGCTTGTCTC-3′; NS2 5′-GGCTGCTGGCACCAGACTTGC-3′) regions were amplified by PCR and sequenced as per Simon et al. (1992)^67^. The ITS and 18S regions were compared against available known sequences in GenBank using BLAST and their classified statuses were determined based on the molecular and morphological data.

### Statistical analysis

Normality and variance constancy were determined for all data. All data were normally distributed. One-way analysis of variance and least significant difference multiple comparison tests were used to determine differences between the analyzed traits of the seedlings in different treatments. Independent t-tests were used to test differences in hormone contents between the PTD37 strain and *T. harzianum* fermentation broth. P-values < 0.05 were considered statistically significant. Compound bar creation and statistical analyses were performed using SPSS 17.0 for Windows (SPSS, Inc., Chicago, IL, USA).
